# Linkage of national soil quality measurements to primary care medical records in England and Wales: a new resource for investigating environmental impacts on human health

**DOI:** 10.1186/s12963-018-0168-2

**Published:** 2018-07-16

**Authors:** Jack E. Gibson, E. Louise Ander, Mark Cave, Fiona Bath-Hextall, Anwar Musah, Jo Leonardi-Bee

**Affiliations:** 1 Division of Epidemiology & Public Health, School of Medicine, University of Nottingham, Clinical Sciences Building Phase II, City Hospital, Hucknall Road, Nottingham, NG5 1PB UK; 2 Centre for Environmental Geochemistry, British Geological Survey, Nicker Hill, Keyworth, Nottingham, NG12 5GG UK; 3 Environmental Geochemistry Baselines Group, British Geological Survey, Nicker Hill, Keyworth, Nottingham, NG12 5GG UK; 4 Centre for Evidence Based Health Care, School of Health Sciences, University of Nottingham, Queen’s Medical Centre, Nottingham, NG7 2HA UK

**Keywords:** Environment and public health [N06], Residence characteristics [N06.850.505.400.800], Catchment area (health) [N06.850.505.400.800.200], Soil [D20.721] [G01.311.820] [N06.230.600], Elements [D01.268], Medical record linkage [E05.318.308.940.968], Epidemiologic methods [N06.850.520], Censuses [N06.850.505.400.225], England [Z01.639.280.300], Wales [Z01.639.280.914]

## Abstract

**Background:**

Long-term, low-level exposure to toxic elements in soil may be harmful to human health but large longitudinal cohort studies with sufficient follow-up time to study these effects are cost-prohibitive and impractical. Linkage of routinely collected medical outcome data to systematic surveys of soil quality may offer a viable alternative.

**Methods:**

We used the Geochemical Baseline Survey of the Environment (G-BASE), a systematic X-ray fluorescence survey of soil inorganic chemistry throughout England and Wales to obtain estimates of the concentrations of 15 elements in the soil contained within each English and Welsh postcode area. We linked these data to the residential postcodes of individuals enrolled in The Health Improvement Network (THIN), a large database of UK primary care medical records, to provide estimates of exposure. Observed exposure levels among the THIN population were compared with expectations based on UK population estimates to assess representativeness.

**Results:**

Three hundred seventy-seven of three hundred ninety-five English and Welsh THIN practices agreed to participate in the linkage, providing complete residential soil metal estimates for 6,243,363 individuals (92% of all current and former patients) with a mean period of prospective computerised medical data collection (follow-up) of 6.75 years. Overall agreement between the THIN population and expectations was excellent; however, the number of participating practices in the Yorkshire & Humber strategic health authority was low, leading to restricted ranges of measurements for some elements relative to the known variations in geochemical concentrations in this area.

**Conclusions:**

The linked database provides unprecedented population size and statistical power to study the effects of elements in soil on human health. With appropriate adjustment, results should be generalizable to and representative of the wider English and Welsh population.

**Electronic supplementary material:**

The online version of this article (10.1186/s12963-018-0168-2) contains supplementary material, which is available to authorized users.

## Background

Soil is a complex mixture of minerals, live and dead organic matter, air, and water. The constituents of soils can enter the human body directly via geophagy or unintentional soil ingestion, airborne dust inhalation and absorption through (or through breaks in) the skin, or indirectly via the food chain (due to prior uptake by crop plants or consumption by livestock). Previous research suggests that adults in developed countries may inadvertently ingest between 23 and 625 mg of soil each day [1]. Soils ubiquitously contain a range of inorganic elements produced through natural soil-forming processes, and as a legacy of inputs from human activity. Some, such as arsenic, cadmium, and lead, have long been recognized as harmful to human health in moderate to high doses [2], although the consequences of long-term low-level exposure remain unclear.

Existing research into the health impacts of soil constituents has tended to focus on geographic areas where abnormally high levels of exposure are observed. Studies of the effects of moderate or trace levels of potentially toxic elements are constrained by the commensurately smaller increases in the risk of adverse health outcomes they may produce. Furthermore, some adverse effects of soil contamination may only become apparent after extended periods of time, even in the presence of high levels of exposure. In consequence, it is often impractical or prohibitively expensive to recruit a sufficiently large (and statistically powerful) population of exposed individuals and to monitor their health over a sufficiently long period for such risks to become detectable.

For this reason, the safe maximum levels of many soil contaminants are not known. Existing official limits are typically based on backwards extrapolation from the known effects of extreme exposures or on inferences drawn from alternative sources of exposure [3].

The ability of epidemiological researchers to detect rare adverse effects of prescribed medications, and to study the causes of rare illnesses has been transformed in recent years by the development of large databases of routinely collected longitudinal data from United Kingdom health care services [4]. The use of such databases permits the construction of virtual study populations, with many years of follow-up, from a pool of millions of individual patients. The range of studies that can be carried out using such databases can be broadened through the linkage of individual patient records to other national data sources based on National Health Service patient identification numbers [5] or on patients’ home addresses [6].

In recent years, the British Geological Survey (BGS) has carried out a comprehensive survey of the inorganic geochemistry of soils throughout England and Wales. We therefore carried out a linkage between these measurements and the medical records of over 7 million current and former patients from 377 primary care practices located throughout England and Wales, based on individuals’ residential postcodes, creating a uniquely large and comprehensive database of soil exposures and health outcomes. To assess the representativeness and generalizability of the linked patient population, we carried out a validation comparing the range of exposures among our patients with those that would be expected based on known population distributions throughout the sampling area.

## Methods

### Data sources

#### BGS Geochemical Baseline Survey of the Environment (G-BASE)

The geochemical data used in this project derive from the BGS systematic national Geochemical Baseline Survey of the Environment (G-BASE) rural and urban soil information [7–9] and from a BGS re-analysis of the National Soil Inventory X-ray fluorescence spectrometry (NSI(XRFS)) samples [10, 11].

**Fig. 1:**
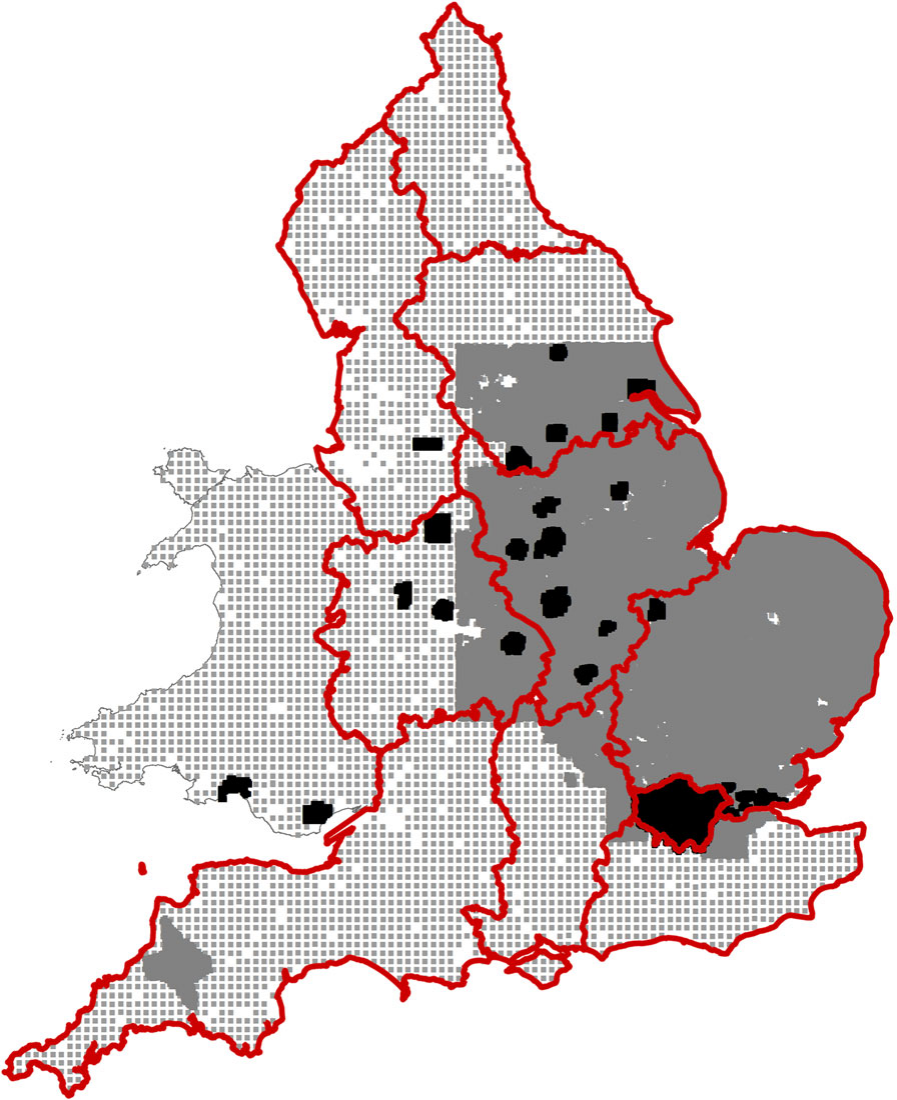
Map of G-BASE sample locations in England and Wales. Black areas denote urban centres where samples are taken at a density of 4 per km^2^. Grey areas denote rural G-BASE areas, with a sampling density of 1 per 2 km^2^. Grey dots indicate locations of NSI(XRFS) samples, at an overall density of 1 per 25 km^2^. Red lines circumscribe the English Strategic Health Authority catchment areas. Contains Ordnance Survey data © Crown Copyright and database rights [2015]

The two projects are closely comparable in their methods of collection: 0–15 cm deep with a sample support of 20 m and subsequently dried at ~ 30 °C sieving to < 2 mm to exclude stones. The most significant difference between these surveys lies in the densities at which samples have been collected and the extent to which these surveys cover the land area of England and Wales. The rural G-BASE (collected from 1968 to 2007) data are concentrated in eastern and central England (Fig. 1) with additional samples from the Tamar catchment of South West England, with samples collected at 1 per 2 km^2^. Urban G-BASE samples have been collected (from 1992 to date) from the centers shown in Fig. 1 at a density of 4 per km^2^, making this the highest density of samples available in this study and the only systematic survey of urban soils in Britain. The NSI(XRFS) samples were collected (initially in the 1980s, with one-third of points resampled in the mid-1990s) at a density of 1 per 25 km^2^ over the whole of England and Wales and provide completeness of coverage of non-urban land areas, albeit at a coarser resolution than the rural G-BASE survey. These merged datasets give a total of 42,422 sample sites with data.

All of these data have been acquired by X-ray fluorescence spectrometry (XRFS) at the BGS laboratories using reference materials that allow bias and precision of the analyses to be monitored, providing a consistency and thus comparability between methods across all three datasets: we therefore jointly refer to these as G-BASE data in the rest of this paper. The use of XRFS is a true total analysis, as it requires no dissolution of the sample [12].

Data are available for 40 elements in total, and of these 15 were selected for linkage with the medical record data on the basis that they are major elements in soil (calcium, aluminium, and silicon), have a role to play in mobility of trace elements (iron [13]) in soil or are trace elements of key interest due to their known or suspected effects on human health (arsenic [14], chromium [15], copper [16], lead [17], manganese [18], nickel [19], phosphorus [20], selenium [21], uranium [22], vanadium [23], and zinc [24]). We excluded cadmium from the final selection, in spite of its known toxicity in humans [25], because the concentrations observed in approximately 75% of G-BASE samples were below XRFS detection limits. Maps showing the variations in concentrations of the included elements throughout England and Wales are available in Additional file 1.

#### The Health Improvement Network database

Each individual in the UK population has the right to register with an NHS primary care practitioner who acts as their first port of call for most non-emergency medical assistance, providing onward referrals to secondary care facilities as necessary. The primary care practitioner is informed of diagnoses made and treatments given or initiated by secondary care institutions as a matter of routine. As a consequence of this gatekeeper role, the primary care medical record provides a comprehensive summary of each patient’s medical history and interactions with the NHS.

The use of electronic medical record (EMR) software packages to create, store, and maintain primary care records has been widespread amongst UK practitioners since the early 1990s and has effectively been mandatory since the introduction in 2004 of the Quality and Outcomes Framework [26], an NHS pay-for-performance scheme which relies on analysis of EMRs to assess compliance with a range of record-keeping and care quality targets. The major EMR packages used by UK practitioners enable offsite backup facilities via a secure electronic link with the software provider.

These characteristics of UK EMR systems, and the relatively small number of packages in use, have facilitated the creation of several health research databases amalgamating data from multiple clinics to create large patient populations. The Health Improvement Network database is one such resource, containing the EMRs of over 10 million patients registered at over 500 practices throughout Great Britain and Northern Ireland, including 450 practices within the G-BASE coverage area. THIN has been extensively used in epidemiological research and the quality of recording has been validated for a wide range of important health outcomes (see e.g., [27–34]).

### Linkage process

In order to preserve patient confidentiality, EMRs in THIN are stripped of details such as names, addresses, days and months of birth, and the exact locations of participating primary care practices prior to their transmission from the practice computer to the THIN data warehouse. Linkage of the G-BASE data to THIN records was therefore carried out within the participating practices themselves.

The G-BASE sample results and sampling locations were loaded into ArcGIS (Esri, Redlands, California, US) and used to interpolate each substance, with an inverse distance weighting (IDW) option with a search radius of 5 km and an output cell size of 1 km. Polygons defining the English and Welsh postcode areas were overlaid, and a summary value (calculated as the concentration intersecting the point of the postcode polygon centroid) determined for each. Where there were no data within the search region, the result was returned as a missing value. Since there are more postcodes (1,526,890) than sample sites, the distance from each postcode polygon centroid to the nearest sample site was calculated to establish the appropriateness of this method of joining the two data sources. This showed that 50% of the postcode centroids were < 1.3 km, and 97% of postcodes < 5 km, from an original sample site. These distances are a close match to the original sampling interval of the survey data (Fig. 1) used to derive the IDW raster surface of concentration values.

These data were output in text format and passed to The Health Improvement Network. Practices falling within the G-BASE coverage area were contacted individually and asked to participate in the linkage exercise. Where consent was given, a copy of the data was uploaded to the practice computer system via a secure electronic link, along with a script to be run by the practice manager. The script searched the G-BASE extract for the postcode of each patient’s current (or last known, for deceased or deregistered patients) residential address and, where a match was found, transmitted the patient’s unique identification code and the summary soil values (but, to preserve confidentiality, not the postcode) back to THIN. The patient identification codes were then used to link the soil values to the anonymized EMRs in the main THIN database.

### Validation

Whilst the exact locations of patients and practices in THIN are unknown to us, the database does provide information on the Strategic Health Authority (SHA) to which each practice belongs. We therefore compared the levels of residential exposure experienced by patients registered in THIN with those that would be expected based on known population distributions in each SHA area.

Expected exposure distributions in each SHA were obtained by first estimating the population of each postcode area. The UK Office for National Statistics (ONS) postcode directory, which gives details of the SHA and census output area (OA) into which each postcode falls, was linked to 2011 OA census populations from the UK data service (formerly UKBorders) [35]. Each postcode within an OA was assumed to contain an equal proportion of the OA population. Where postcodes crossed an OA or SHA border, the postcode was randomly assigned to a single area.

The postcode-specific population estimates were then linked to the postcode-specific soil values obtained previously, and boxplots describing the expected exposure distributions in each SHA area were generated. Comparison boxplots were constructed describing exposures among all THIN patients who were alive and actively registered on the date of the 2011 census.

## Results

### Practice participation and patient coverage

At the time the data linkage process commenced, THIN contained information from 450 primary care practices in England and Wales. Of these, 395 were active contributors to THIN at the time the linkage commenced, with the remainder having left, merged, or closed at some point prior. 377 (95%) of active practices agreed to participate in the linkage. The participation rate among practices in each SHA area is shown in Table 1. Participation was greatest in the West Midlands and South Central SHAs (both 100%) and lowest in Yorkshire & Humber (78%). The conspicuously low rate in the latter case in fact represents only two refusals, the low percentage being a consequence of the small number of THIN practices in this region.

**Table 1:** Number of practices participating in the linkage between The Health Improvement Network database and the Geological Baseline Survey of the Environment, by Strategic Health Authority area

Strategic Health Authority	Participating practices	Active practices	Participation rate (%)
East Midlands	12	13	92
East of England	26	30	87
London	59	61	97
North East	12	13	92
North West	52	54	96
South Central	47	47	100
South East Coast	41	43	95
South West	45	46	98
Wales	36	39	92
West Midlands	40	40	100
Yorkshire & Humber	7	9	78
Total	377	395	95

At the time of the linkage, THIN contained the medical records of 7.1 million patients currently or formerly registered at the 395 actively contributing practices. The average period of prospective computerized data recording (follow-up) was 6.75 years. 6.8 million (96%) of these patients were registered at a practice that agreed to participate in the linkage. 6.3 million patients (93%) from linkage practices lived in a postcode where at least one soil element level could be obtained from G-BASE, and 6.2 million patients (92%) lived in an area where all 15 were available.

Of the patients with no soil measurements following the linkage process, 144,600 lived in a postcode listed in the postcode file uploaded to the practices, but for which there was no G-BASE coverage. For the remainder, the matching process failed entirely, indicating that the affected patients either had no postcode recorded in their medical records, an invalid or out-dated postcode, or a valid postcode not listed in the file uploaded to the practices (possibly because the postcode was newly-created).

Table 2 shows the matching levels broken down by SHA. The percentages of patients registered at linkage practices were similar to the percentages of practices that participated in the linkage (Table 1) suggesting that there was no marked difference in the sizes of the practices that declined to take part. The matching process was most successful in the East Midlands and the East of England, with 96 and 97% of patients respectively having the complete set of G-BASE measures, and least successful in London and the North East (84% complete in both cases.

**Table 2:** Proportions of all patients ever registered at practices actively contributing data to The Health Improvement for whom partial (at least one element) or complete (all elements) soil measurements were obtained through linkage to the Geochemical Baseline Survey of the Environment (G-BASE)

Strategic Health Authority	Total patients at active practices	Patients at linkage practices (% of total)	Patients with partial G-BASE data (% of patients at linkage practices)	Patients with complete G-BASE data (% of patients at linkage practices)
East Midlands	212,395	198,085 (93.3%)	190,681 (96.3%)	190,679 (96.3%)
East of England	574,828	474,517 (82.5%)	462,008 (97.4%)	462,005 (97.4%)
London	1,208,044	1,190,099 (98.5%)	1,000,246 (84.0%)	1,000,232 (84.0%)
North East	218,821	208,238 (95.2%)	175,302 (84.2%)	175,301 (84.2%)
North West	767,722	733,446 (95.5%)	660,898 (90.1%)	656,475 (89.5%)
South Central	1,069,029	1,069,029 (100.0%)	1,040,055 (97.3%)	1,035,340 (96.8%)
South East Coast	873,765	845,650 (96.8%)	804,428 (95.1%)	804,367 (95.1%)
South West	763,658	752,735 (98.6%)	706,818 (93.9%)	706,817 (93.9%)
Wales	564,190	503,432 (89.2%)	485,619 (96.5%)	451,544 (89.7%)
West Midlands	716,592	716,592 (100.0%)	662,649 (92.5%)	633,347 (88.4%)
Yorkshire & Humber	168,321	133,559 (79.3%)	131,448 (98.4%)	127,256 (95.3%)
Total	7,137,365	6,825,382 (95.6%)	6,320,152 (92.6%)	6,243,363 (91.5%)

The complete THIN database contains the details of all patients for whom an EMR has ever been created at a participating practice, even in cases where the patient died or deregistered some time ago. In order to carry out a like-for-like comparison between soil measurements in THIN and expectations based on the population distribution at the time of the 2011 census, it was necessary to restrict to patients alive and actively registered on this date. Among these patients the matching process was more successful, reflecting the increased likelihood that practitioners will hold a correct, up-to-date postcode for current or recent patients. 3.2 million patients were registered at actively participating practices. 3.0 million (96%) were registered at a linkage practice. Complete and partial G-BASE measurements were available for 95% (2.9 million) and 96% (2.9 million) of these patients respectively.

Sixty-six thousand three hundred twenty-seven patients lived in an area with no G-BASE coverage and 62,054 patients could not be matched. Table 3 shows the matching levels broken down by SHA. Again, the proportions of patients matched were similar to practice participation rates. The linkage was most successful in the East Midlands and the East of England (almost 100% of patients having complete G-BASE data in both areas), and weakest in the North East (88% complete) and London (89% complete).

**Table 3:** Proportion of patients alive and registered on the date of the 2011 census at practices actively contributing data to The Health Improvement Network for whom partial (at least one element) or complete (all elements) soil measurements were obtained through linkage to the Geochemical Baseline Survey of the Environment (G-BASE)

Strategic Health Authority	Total patients at active practices	Patients at linkage practices (% of total)	Patients with partial G-BASE data (% of patients at linkage practices)	Patients with complete G-BASE data (% of patients at linkage practices)
East Midlands	102,970	96,498 (93.7%)	96,448 (99.9%)	96,448 (99.9%)
East of England	262,070	219,216 (83.6%)	219,038 (99.9%)	219,038 (99.9%)
London	443,050	436,073 (98.4%)	388,973 (89.2%)	388,970 (89.2%)
North East	105,225	99,672 (94.7%)	87,656 (87.9%)	87,656 (87.9%)
North West	375,752	361,339 (96.2%)	335,503 (92.8%)	333,132 (92.2%)
South Central	469,322	469,322 (100.0%)	467,804 (99.7%)	466,069 (99.3%)
South East Coast	388,040	369,537 (95.2%)	362,703 (98.2%)	362,676 (98.1%)
South West	357,656	353,201 (98.8%)	337,712 (95.6%)	337,711 (95.6%)
Wales	267,513	242,972 (90.8%)	240,679 (99.1%)	225,442 (92.8%)
West Midlands	333,920	333,920 (100.0%)	316,876 (94.9%)	303,968 (91.0%)
Yorkshire & Humber	81,111	63,283 (78.0%)	63,260 (100.0%)	61,119 (96.6%)
Total	3,186,629	3,045,033 (95.6%)	2,916,652 (95.8%)	2,882,229 (94.7%)

### Comparison between the THIN population and the overall population

Additional file 2 shows the results of the comparison between the observed levels of the linked measurements among the THIN population and those expected among the wider population of England and Wales. Overall, the patterns are very similar, with the most marked differences largely restricted to the Yorkshire & Humber SHA, which has the both smallest number of participating practices and the smallest patient population. In particular, this region exhibits comparatively restricted ranges of exposures to arsenic, chromium, iron, manganese, and phosphorus.

## Discussion

### Key findings

By linking, at postcode level, the G-BASE and THIN databases, we were able to obtain residential soil element levels for patients at 95% of English and Welsh practices, with complete geochemical data being available for 92% of patients within those practices. The levels associated with patients in the THIN database are in line with expectations based on the known population distributions in England and Wales, except in areas where there are few THIN practices.

### Strengths and limitations

To our knowledge, the new linked resource is unique, providing unprecedented population size and statistical power to study the effects of elements in soil on human health. The data provide comprehensive, prospective recording of health outcomes across a population of over 6 million individuals offering, in principle, the potential to study the effects (whether adverse or beneficial) of any soil constituent present in the linked dataset on the risk of any medical condition diagnosed by or reported to primary care practitioners.

The additional health care and lifestyle details recorded in EMRs provide us with the ability to adjust for a wide range of potential confounding factors which may cluster geographically, as do the prior linkages of the THIN database to measures of area-level socioeconomic status, air pollution, and land use. The wide range of soil constituent measures we have linked will permit adjustment for the presence of other elements which may also modify the risk of outcomes of interest, and enable us to assess the extent of effect modification due to the presence of elements which may affect bioavailability (as in the case of iron and arsenic) [13].

The similarity of the soil constituent exposure levels observed among THIN patients to those that would be expected in the wider population suggests that studies using the linked resource are likely to produce generalizable results. Previous validation studies of the THIN database indicate that participants are representative of the population at large in terms of a range of sociodemographic measures [36].

There are a number of limitations that may affect the utility of the linked database in practice. The sampling resolution of the surveys in G-BASE may conceal focal areas of high variability in soil constituents. Local heterogeneity is generally greater in urban areas, but this is superimposed upon systemically increased concentrations associated with the impact of urbanisation for elements such as lead and copper [37, 38]. In urban areas, where the THIN population is concentrated, sampling density is high (4 per km^2^) and work carried out during the completion planning for G-BASE suggests that improvements in estimate precision above the 1 per 2 km^2^ level may be relatively small [39], although this will vary from element to element.

Uncertainties always exist in the interpolation of values between points of measured concentration to make predictions at unsampled locations. We used the inverse distance weighting method as it is a relatively straightforward and widely understood approach that produces estimates primarily determined by the closest available sample site. Point estimates at the postcode centroid (rather than an alternative such as an average of all points within a postcode) were considered sufficient as UK postcode areas are small (especially relative to the distance between sampling sites): in urban areas each typically represents a small section of a street, or even a single large apartment building) and contains an average of 15 (range 1–100) individual mail delivery addresses [40]. More sophisticated techniques (such as those based on machine learning) which incorporate information from additional mapping layers have been shown to improve precision in subsets of the G-BASE data [41], however this is an ongoing area of research and such methods have not yet been applied or validated across the full survey area.

We cannot be certain that the presence of raised levels of a contaminant in the soil in the area where each patient lives directly translates into increased exposure among those patients; where patients work a long distance from home, consume little locally-produced produce, seldom engage in outdoor activities such as sports or gardening, or live in focal areas of severe contamination, the true exposure level may be markedly different. The presence of a substantial number of such individuals in the THIN population would tend to introduce random error. This would typically manifest as a null bias, so whilst it is unlikely to lead to the false identification of an increased risk, the magnitude of a true risk might be underestimated. The large size of the THIN population (and concomitant statistical power) will reduce the impact of such bias on our ability to detect raised risks, even in cases where we are unable to accurately quantify them.

The participation rate among practices in the Yorkshire and Humber SHA was low, which may restrict our ability to draw inferences about the risks experienced by patients in this area. In addition, there is a known bias towards arable land within the NSI(XRFS) sample collection areas (the survey was initially carried out to help assess agricultural potential). This issue primarily affects West Wales, where known examples of industrial land contamination are not detectable in the NSI(XRFS) dataset [37, 42]. We are unable to distinguish between different compound forms of the elements included in the linkage, which may be problematic where toxicity or effects on bioavailability differ [43]. For example, different forms of iron are known to differentially affect the bioavailability of arsenic in soils [13]. It is likely, however, to be possible to at least partially adjust for this at area level; whilst we do not know the exact locations of patients or practices in the linked dataset, we do know to which Strategic Health Authority area each practice belongs, and the ratios between ironstones and other mineral forms of iron differ substantially between these areas [44].

Whilst the THIN data are longitudinal, the G-BASE data are (although collected over an extended period) effectively cross-sectional, and the linkage has been carried out at a single point in time. The exposure levels assigned to each individual may not, therefore, be representative over the entire duration of follow-up. Previous research suggests that levels of most of the soil constituents included in the linkage are driven by (generally slow) geological processes and that levels are relatively stable over time, except in areas and for elements where there are significant ongoing inputs from industrial or agricultural activities [45].

The linked measures are unlikely to accurately reflect long-term exposure for patients who have only been registered for a short time, however it should be possible to address this issue by carrying out sensitivity analyses restricted to patients who have been continuously registered for an extended period. In addition, THIN is updated quarterly, so the duration of follow-up available for the patients included in the linkage will increase over time. Movement of patients away from (and registration of new participants into) participating practices will, over time, reduce the proportion of patients for whom soil measures are available, requiring the linkage to be repeated. The patients who leave the database will be more likely to be those who are in highly mobile sociodemographic groups, somewhat reducing the demographic representativeness of the linked population, but at the same time preferentially removing those for whom point estimates of exposure are least likely to reflect lifetime exposure.

When linking geospatial and medical datasets there is, in each case, a need to make compromises in order to preserve patient confidentiality. The THIN/G-BASE linkage demonstrates a viable approach that provides high quality, individual-level data on a very large number of patients at the cost of limiting our knowledge of patient locations and the number of geochemical variables we were able to link (to avoid producing unique combinations which would make postcodes and patients readily identifiable). It is unlikely that linkages providing spatial information in sufficient detail for risk mapping and GIS analysis, or that incorporate richer information about soils (e.g., more constituents, or details of other soil characteristics that may influence exposure or bioavailability) would receive ethical approval in most jurisdictions unless either explicit patient consent was obtained (limiting the feasibility of assembling a large research population), or summary data on population health was used in place of individual patient records. This situation may improve in the near future, however, as emerging techniques for secure multi-party statistical analysis [46] may enable multiple data-holders to carry out rich joint analyses without explicitly linking or sharing their datasets with one another and creating confidentiality concerns in the process.

### Arrangements for access

Given the wide potential scope for studies using the linked database, it is our hope that external researchers will make use of it in their research and we have put in place a process to enable wider access. Both the THIN and G-BASE components of the linked data are subject to licensing restrictions, and ethical approval is required from the THIN Scientific Research Committee before data extracts can be made available. Parties interested in obtaining data for research projects should contact IQVIA (https://www.iqvia.com/locations/uk-and-ireland/thin) in the first instance.

## Conclusions

The linkage of millions of primary care electronic medical records of patients throughout England and Wales to individual-level estimates of residential soil element exposure opens new avenues for research in environmental public health, providing a cohort with considerable statistical power to investigate even minor effects across an extremely wide range of health outcomes. Our findings suggest that, with appropriate adjustment, results should be generalizable to and representative of the wider English and Welsh population.

## Additional files

Additional file 1: Maps of soil element levels by United Kingdom Strategic Health Authority area. Contains Ordnance Survey data © Crown Copyright and database rights [2015]. (DOCX 33519 kb)

Additional file 2: Observed range of concentrations of 15 linked constituent elements in the residential soils of patients enrolled in The Health Improvement Network database on the date of the 2011 UK census, by Strategic Health Authority, and comparison with the expected range of concentrations in the residential soils of the entire Strategic Health Authority populations, estimated using 2011 Census population distributions. Boxes indicate interquartile ranges, midlines indicate median values and whiskers are drawn to the upper and lower adjacent values. (PDF 87 kb)

## Abbreviations

BGS: The British Geological Survey, United Kingdom
EMR: Electronic medical record
G-BASE: The British Geological Survey systematic national Geochemical Baseline Survey of the Environment
NHS: The United Kingdom National Health Service
NSI (XRFS): The National Soil Inventory X-Ray fluorescence spectrometry sample set
OA: United Kingdom census 2011 output area
SHA: (English) Strategic Health Authority
THIN: The Health Improvement Network database
UK: The United Kingdom of Great Britain and Northern Ireland
XRFS: X-ray fluorescence spectrometry
