# Supplementary material for: Linkage of national soil quality measurements to primary care medical records in England and Wales: a new resource for investigating environmental impacts on human health
Source: Popul Health Metr. 2018 Jul 16;16:12. doi: 10.1186/s12963-018-0168-2 (PMC6048879; doi:10.1186/s12963-018-0168-2)

# Linkage of national soil quality measurements in England and Wales to primary care medical records: a new resource for investigating environmental impacts on human health.

Jack E Gibson^1^, E Louise Ander^2a^, Mark Cave^2b^, Fiona Bath-Hextall^3^, Anwar Musah^1^, Jo Leonardi-Bee^1^.

1. Division of Epidemiology & Public Health, School of Medicine, University of Nottingham, UK.
2. ^a^Centre for Environmental Geochemistry and ^b^Environmental Geochemistry Baselines Group, British Geological Survey, Keyworth, UK.
3. School of Health Sciences, University of Nottingham, UK.

Figure S1 – Geographic variations in concentrations of elements included in the linkage between The Health Improvement Network database and the British Geological Survey Geochemical Baseline Survey of the Environment, England and Wales. Red borders show the boundaries of the 10 English Strategic Health Authorities. Grey borders show the boundaries of local health authorities. Panel A – Aluminuim; B – Arsenic; C – Calcium; D – Chromium; E – Copper; F – Iron; G – Lead; H – Manganese; I – Nickel; J – Phosphorus; K – Selenium; L – Silicon; M – Uranium; N – Vanadium; O – Zinc.

A
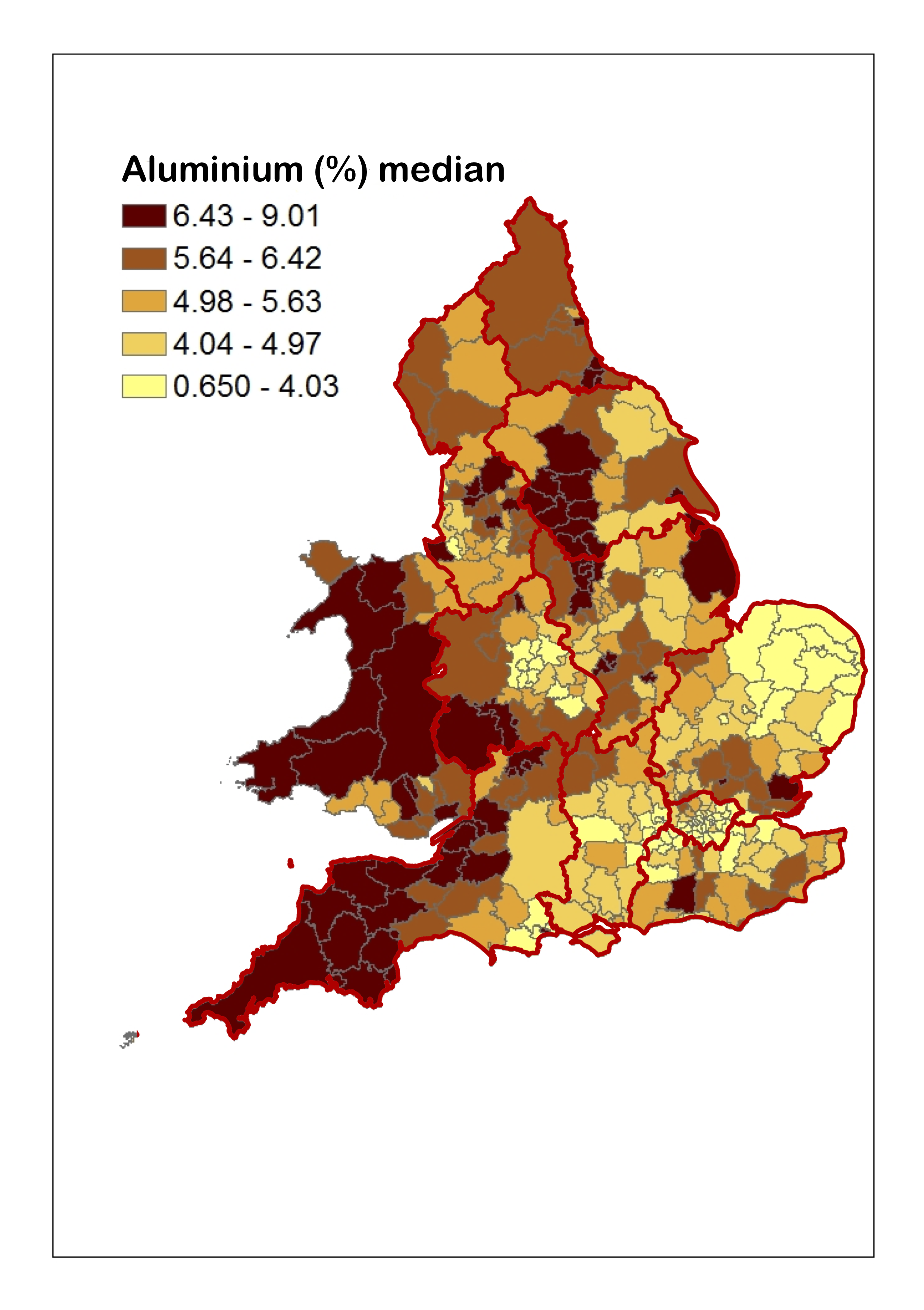
B
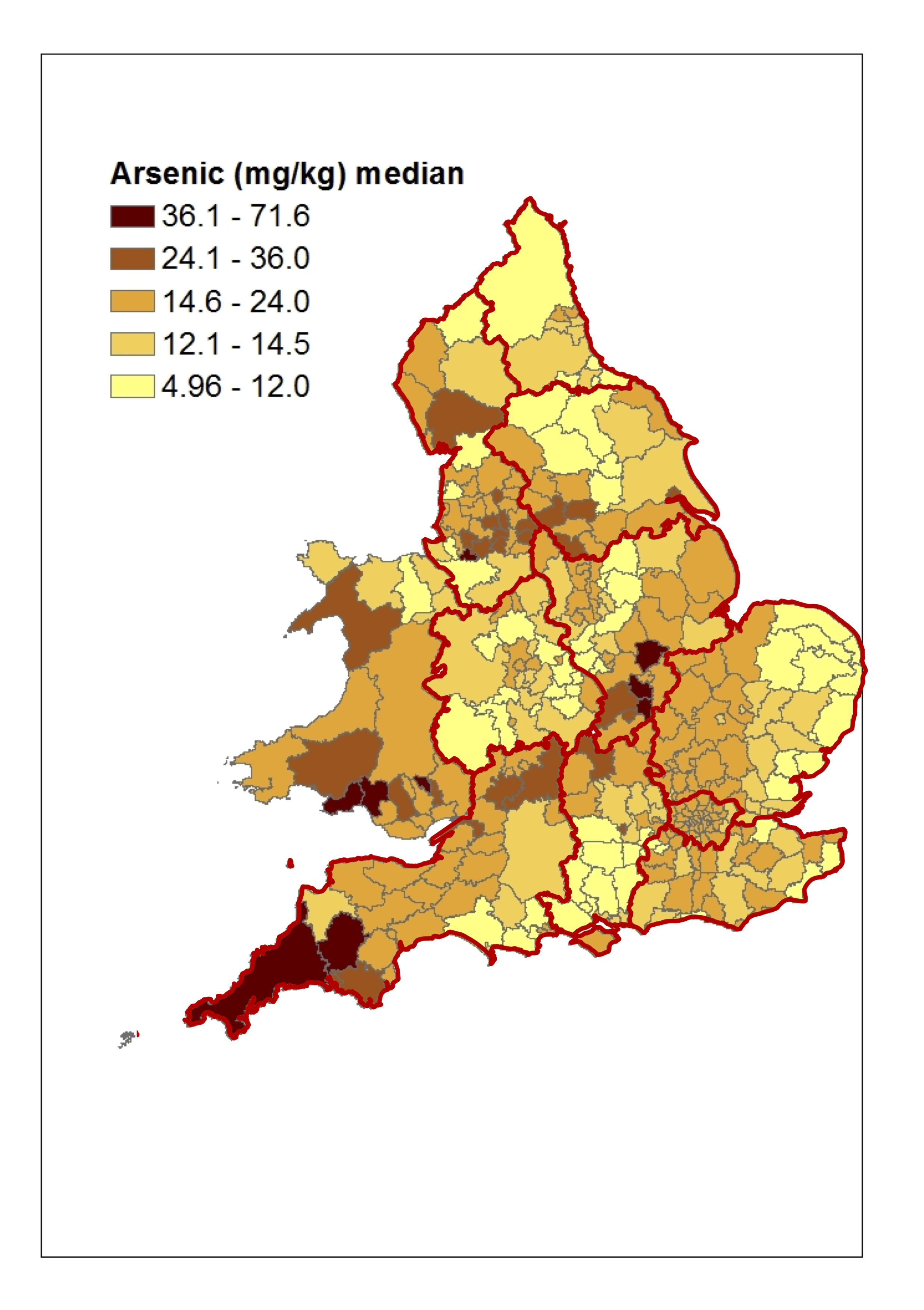


C
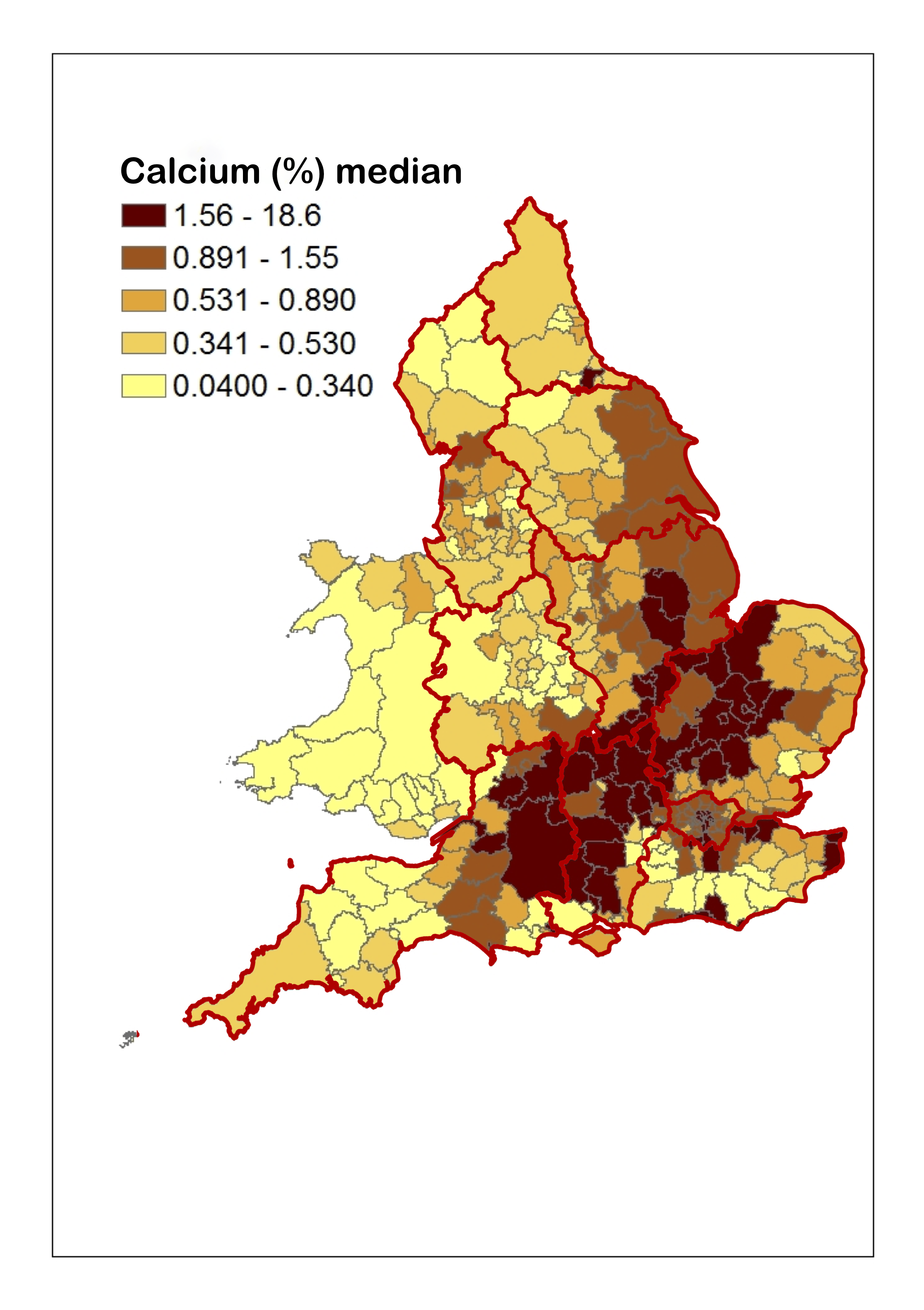
D
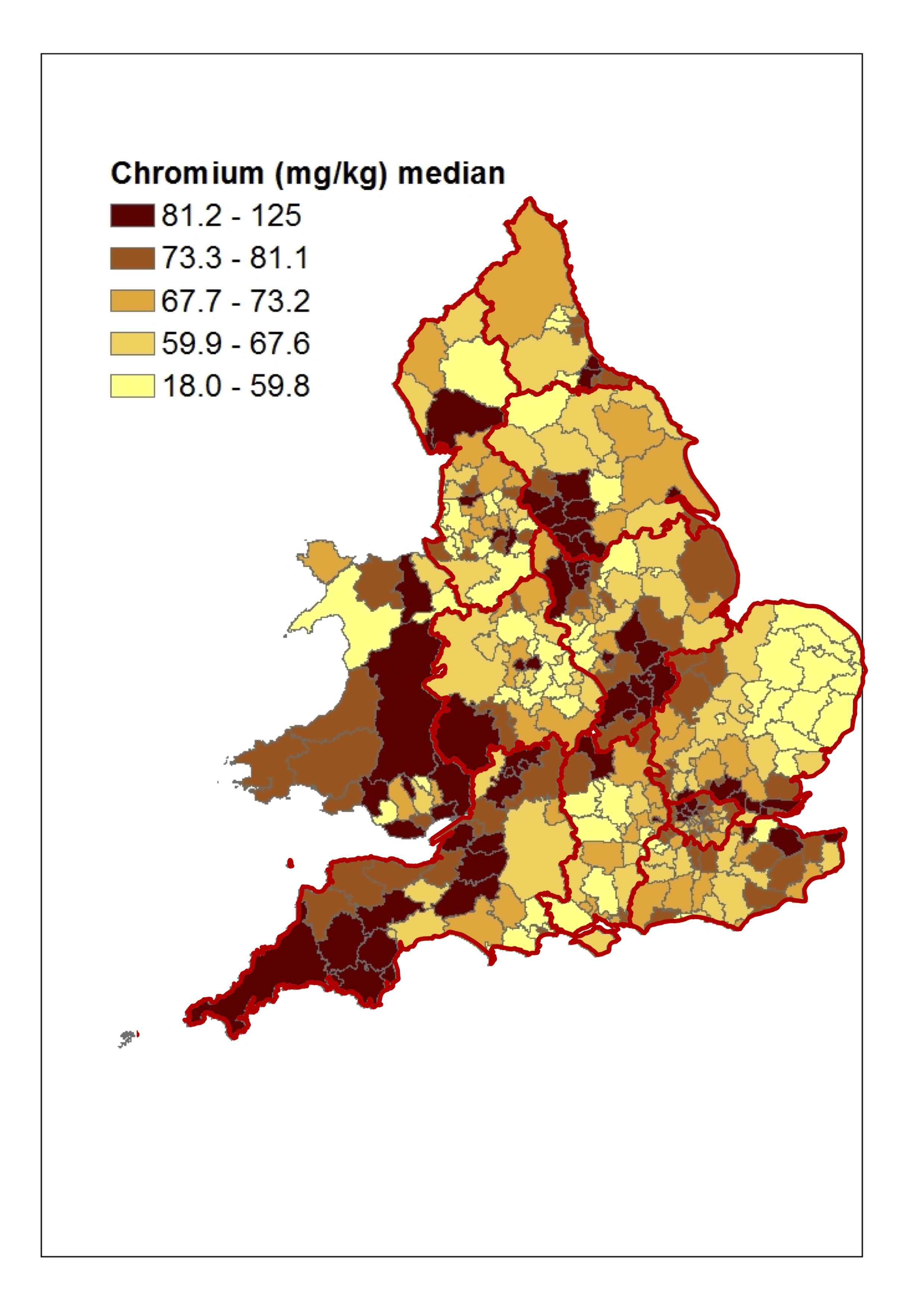


E
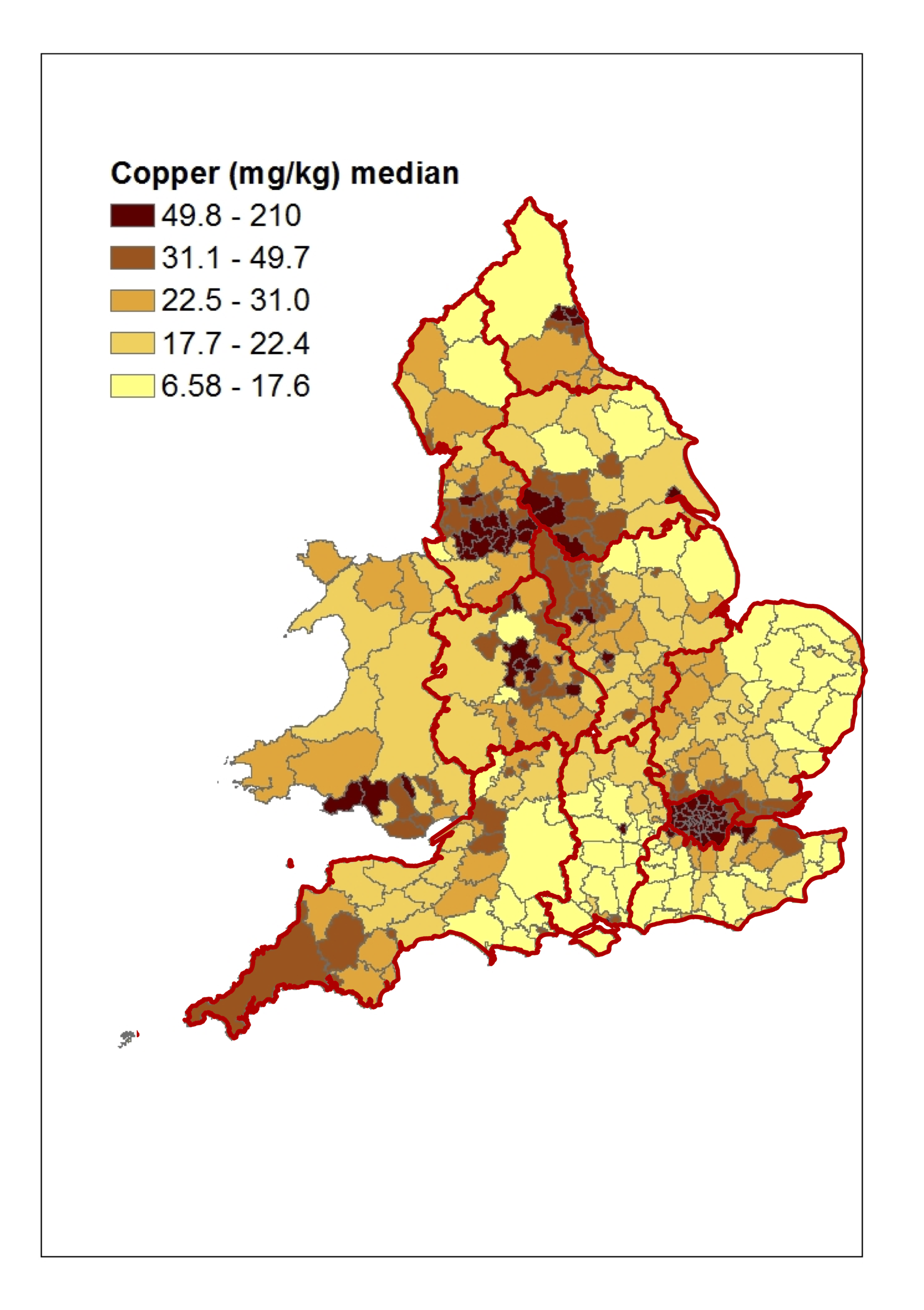
F
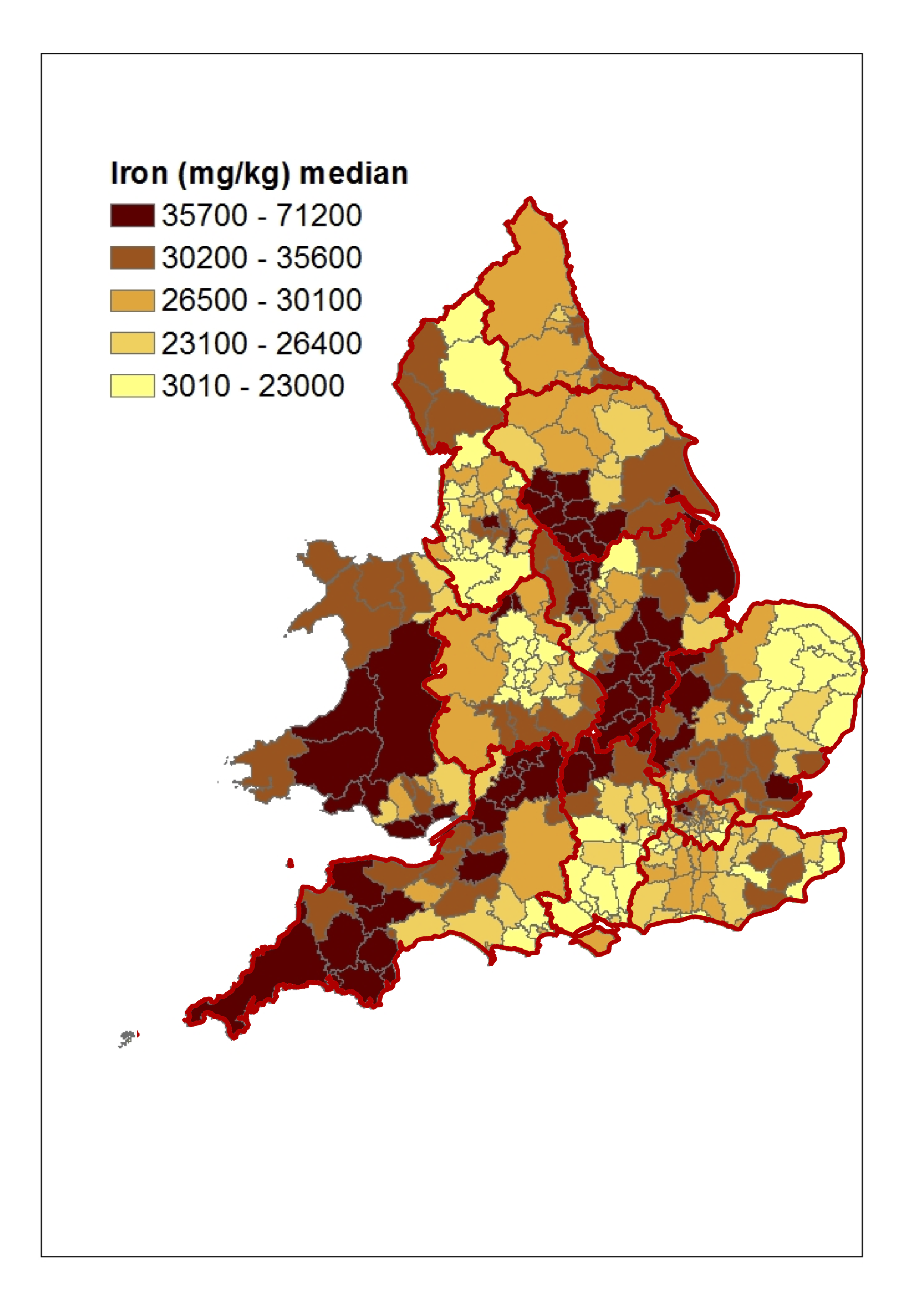


G
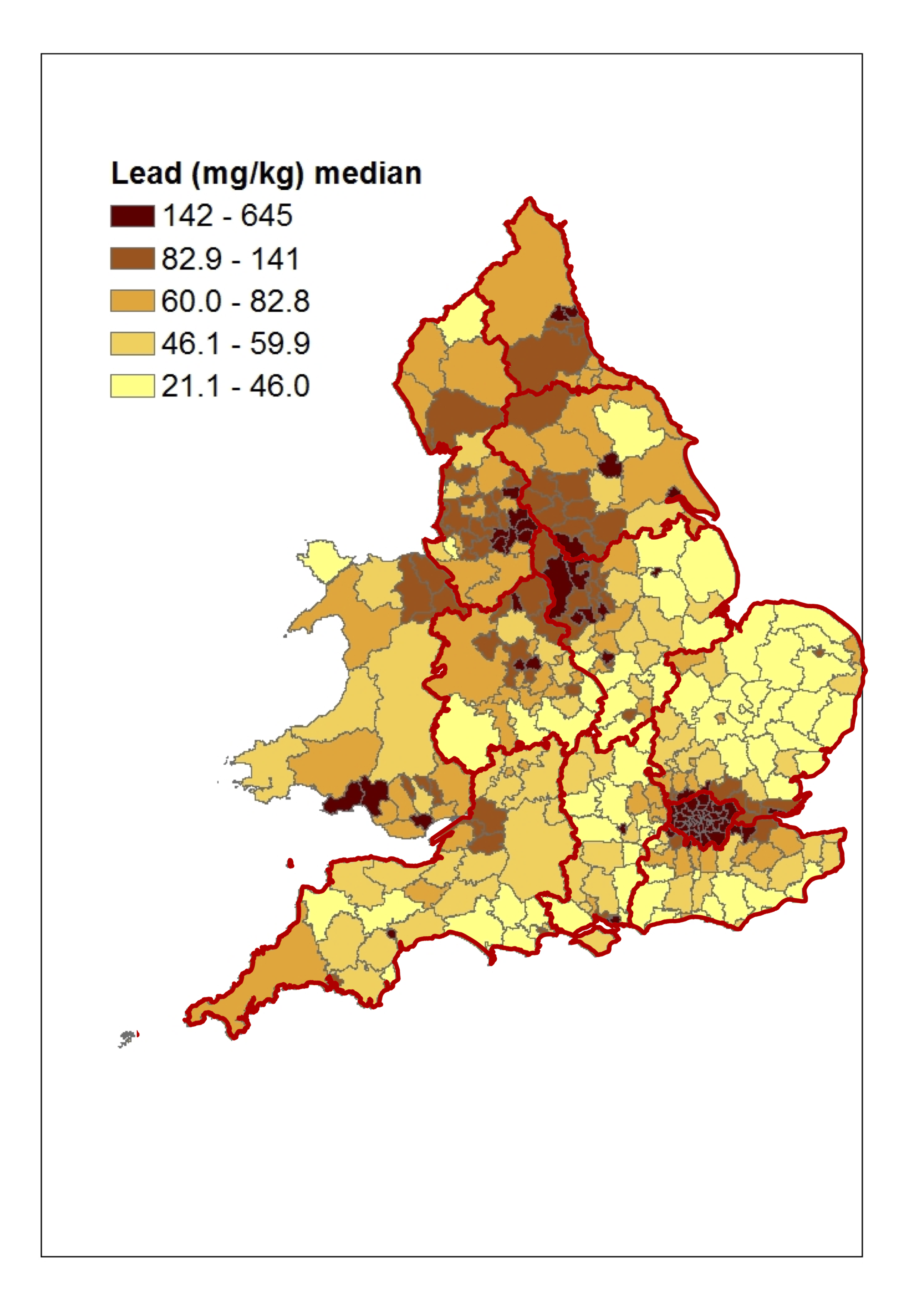
H
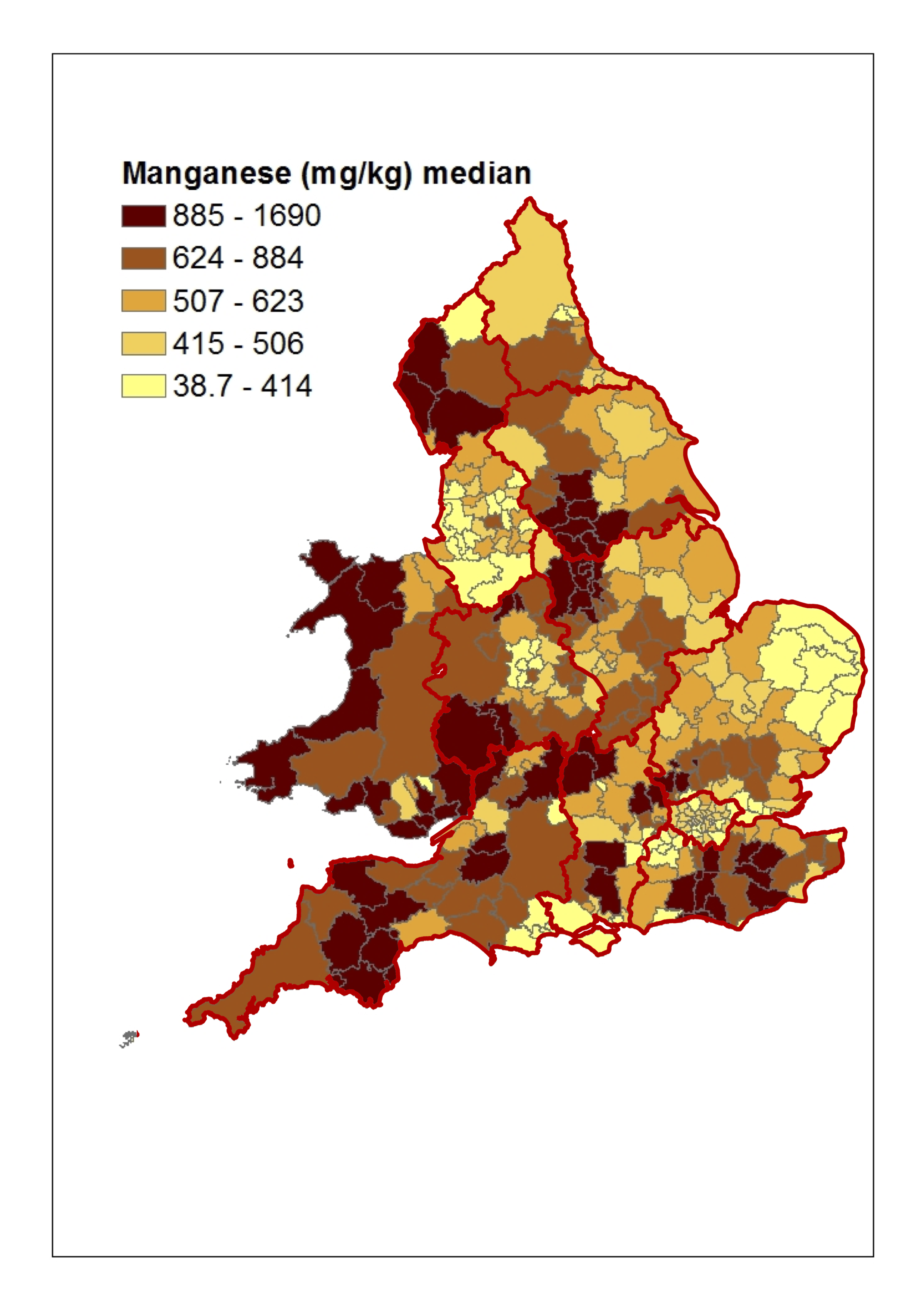


I
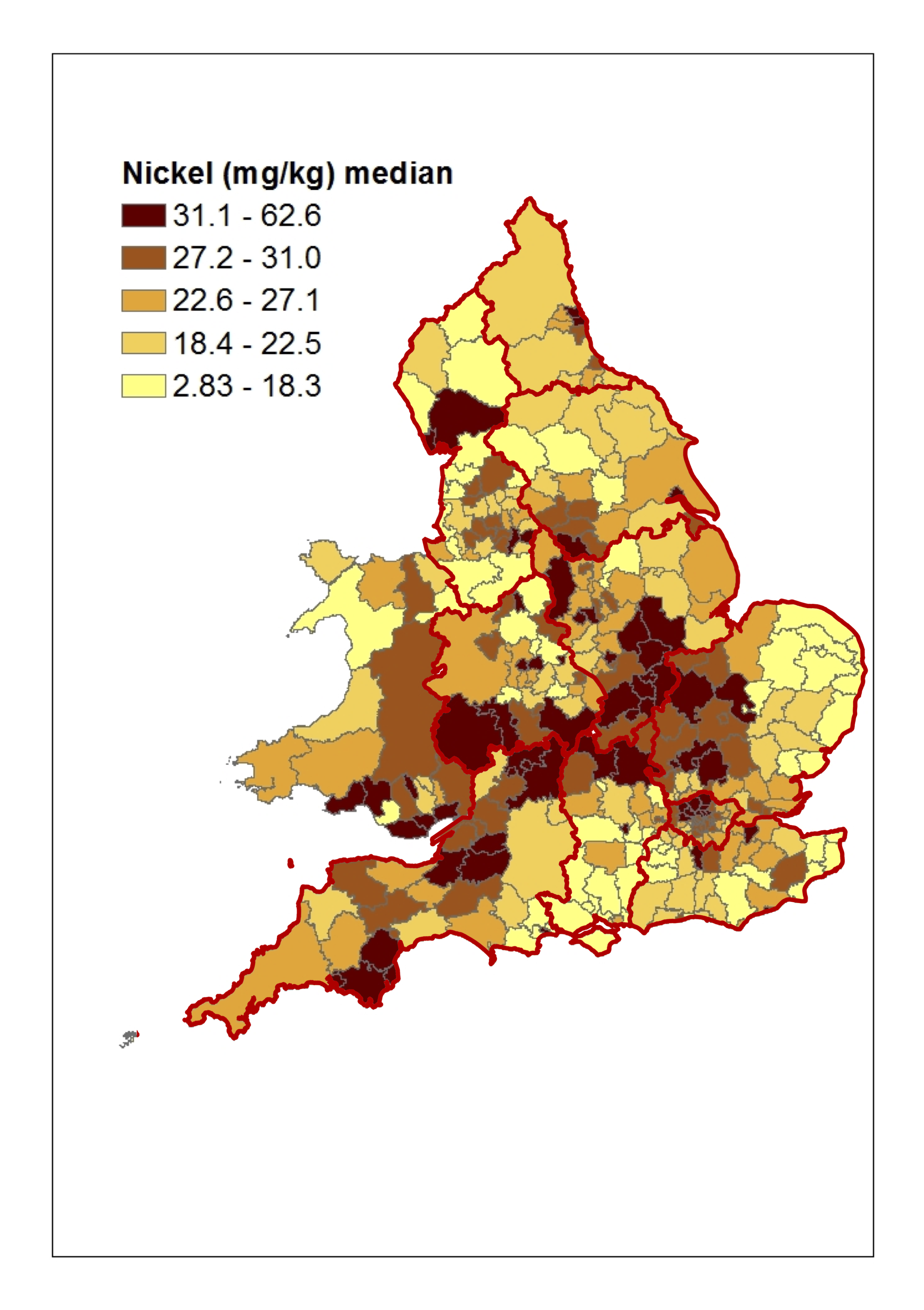
J
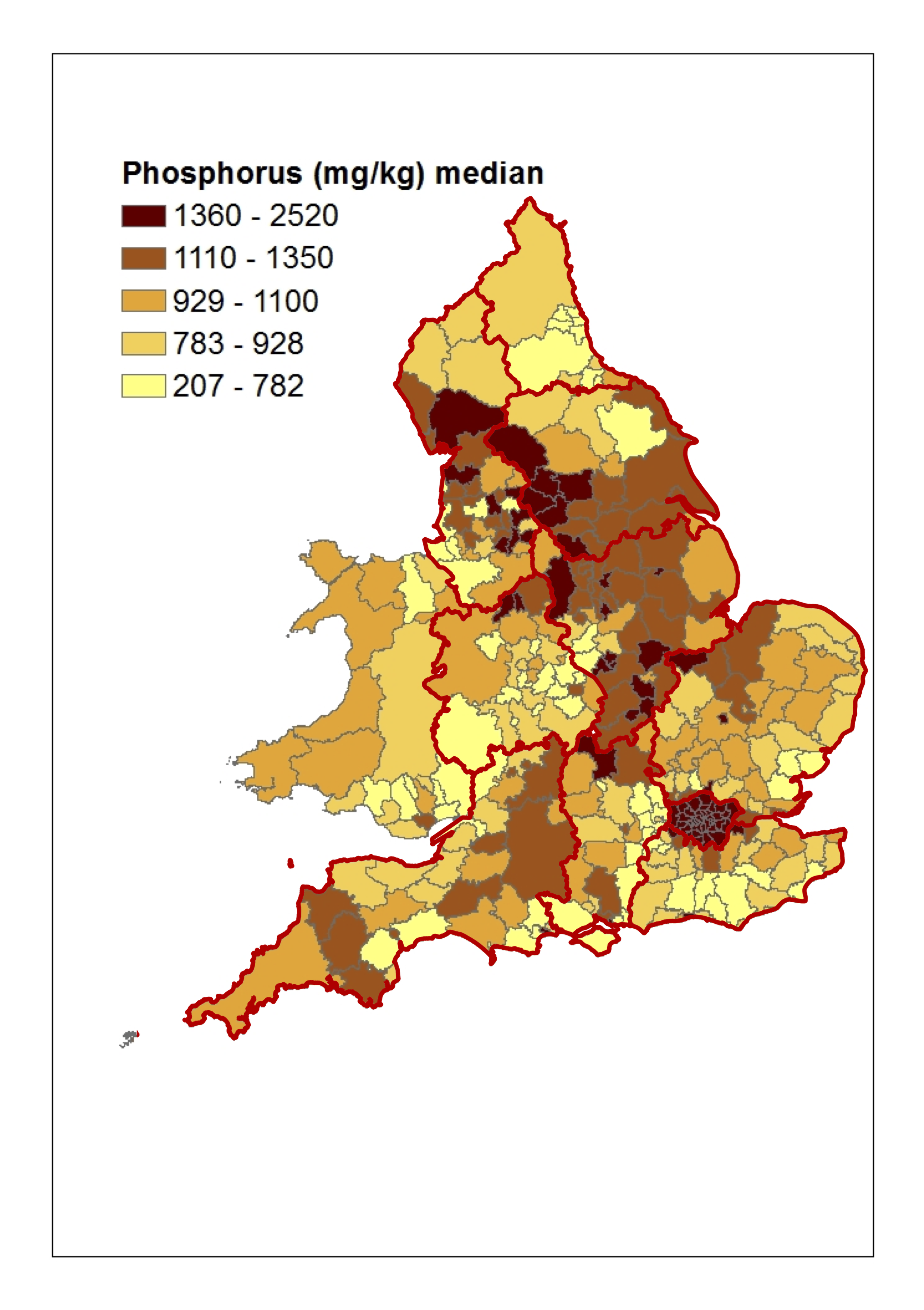


K
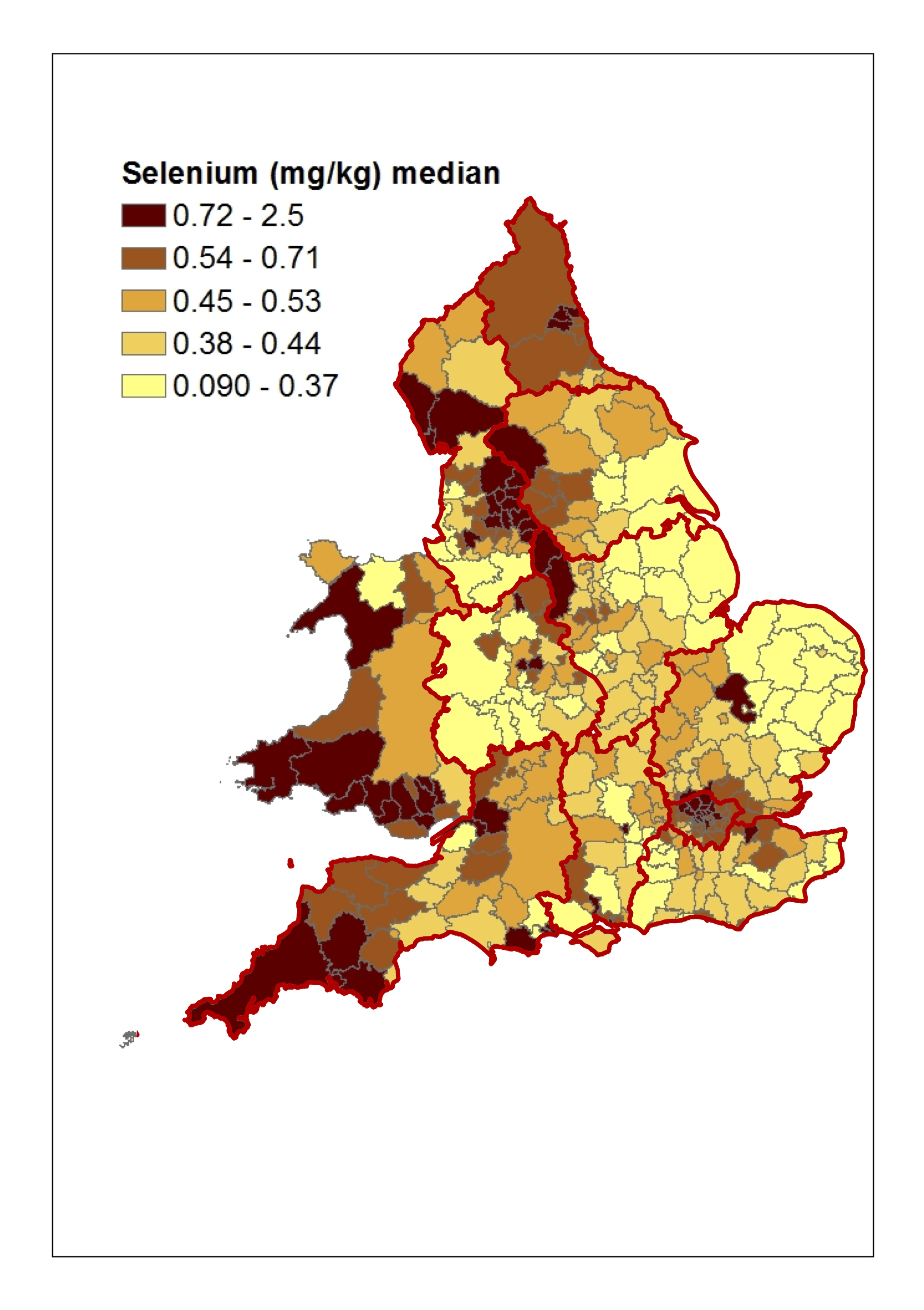
L
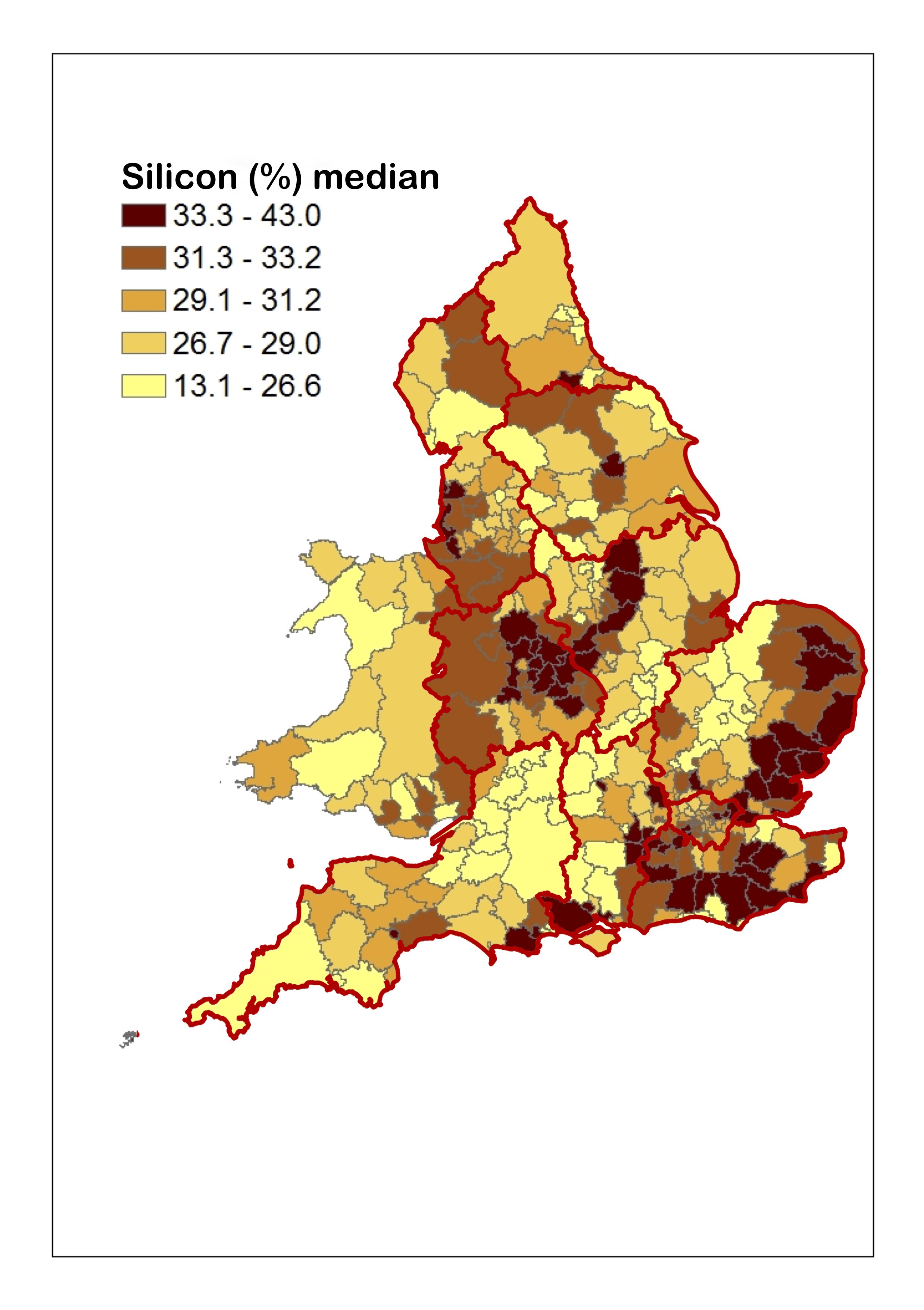


M
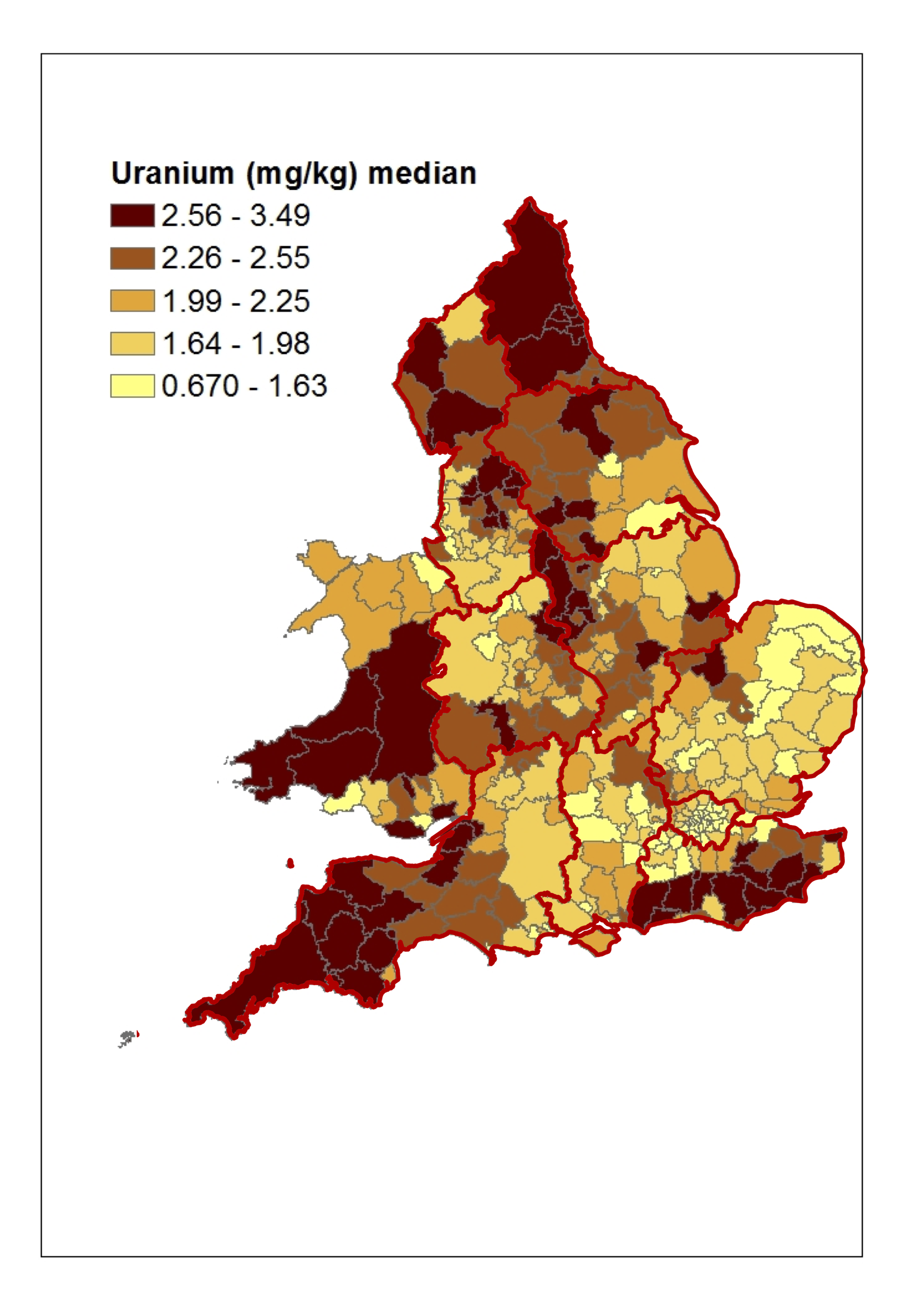
N
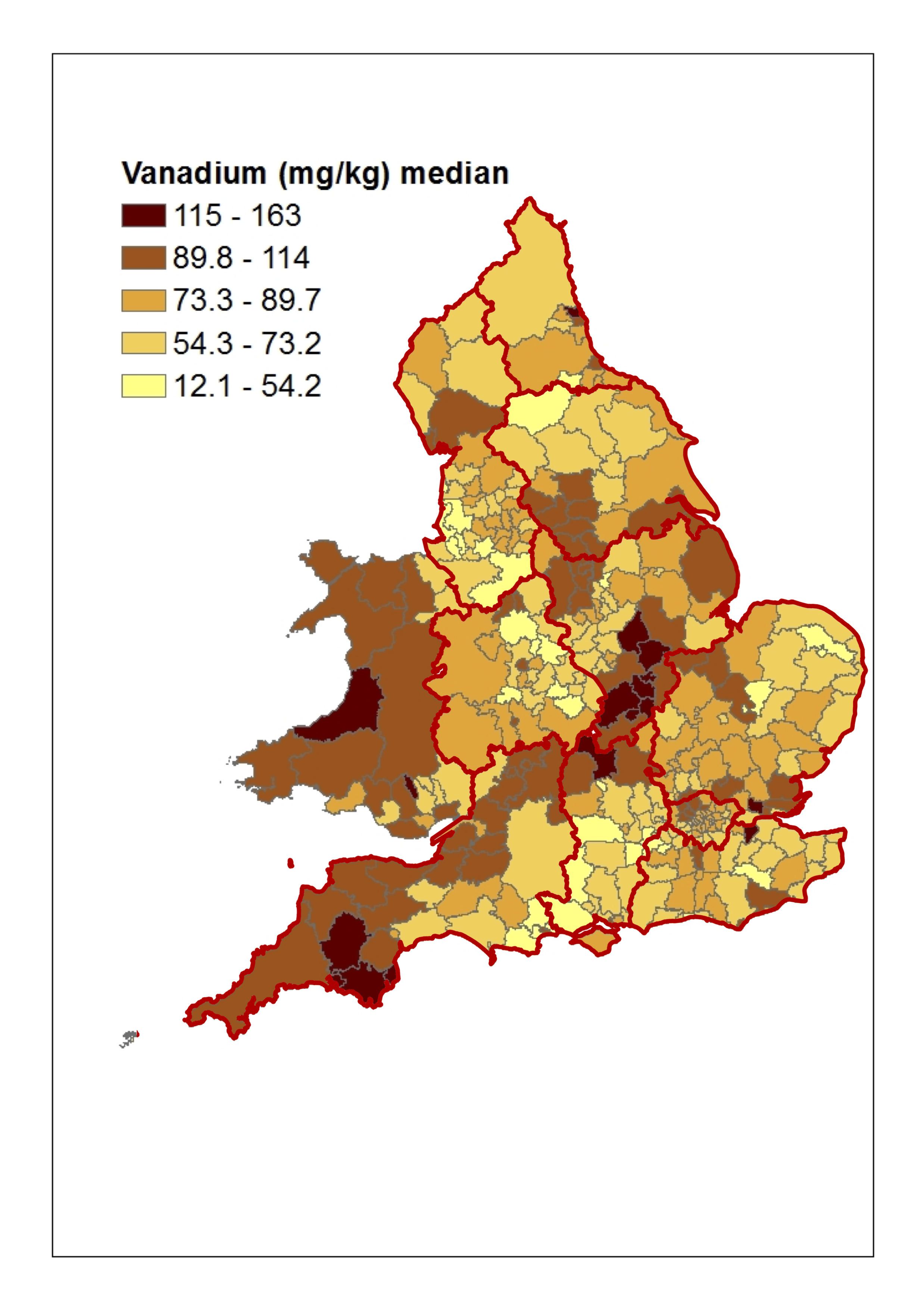


O
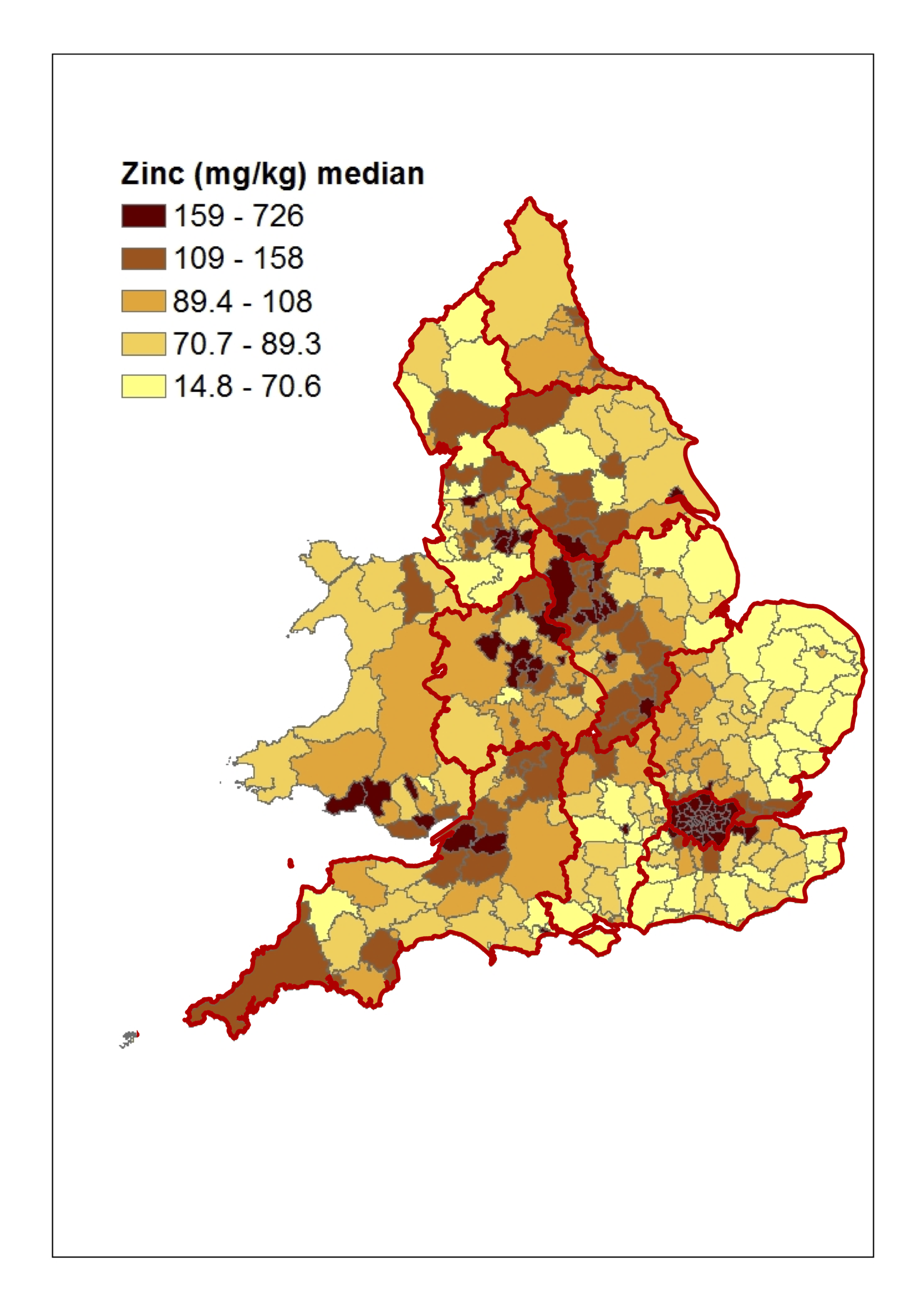

Supplement: Supplementary file 1 — Maps of soil element levels by United Kingdom Strategic Health Authority area. Contains Ordnance Survey data © Crown Copyright and database rights [2015]. (DOCX 33519 kb) [file 12963_2018_168_MOESM1_ESM.docx]
