# Supplementary figures and images for: Linkage of national soil quality measurements to primary care medical records in England and Wales: a new resource for investigating environmental impacts on human health
Source: Popul Health Metr. 2018 Jul 16;16:12. doi: 10.1186/s12963-018-0168-2 (PMC6048879; doi:10.1186/s12963-018-0168-2)

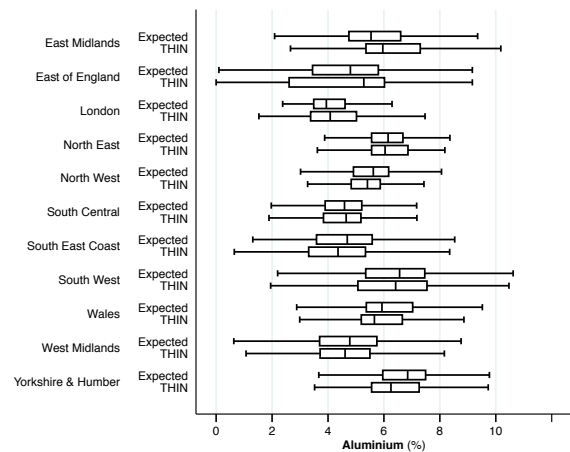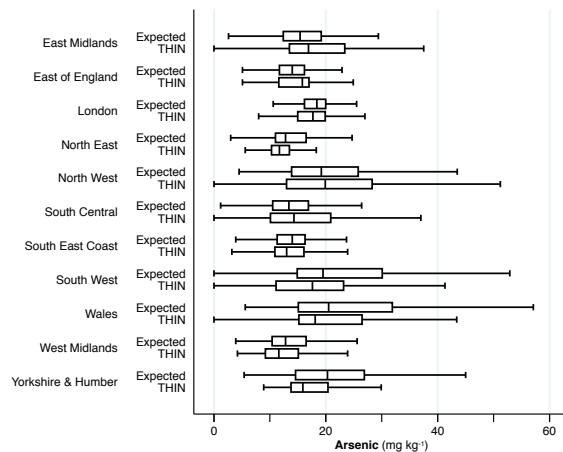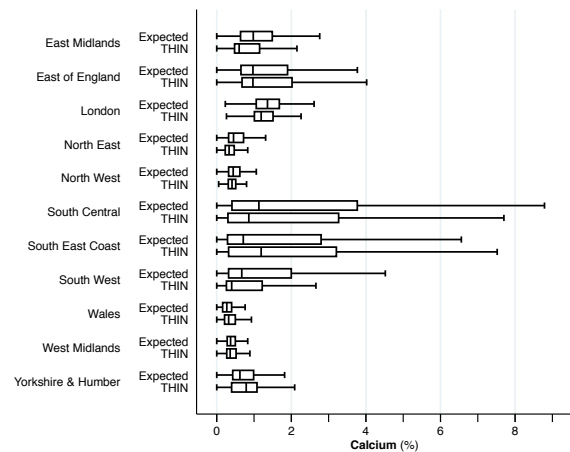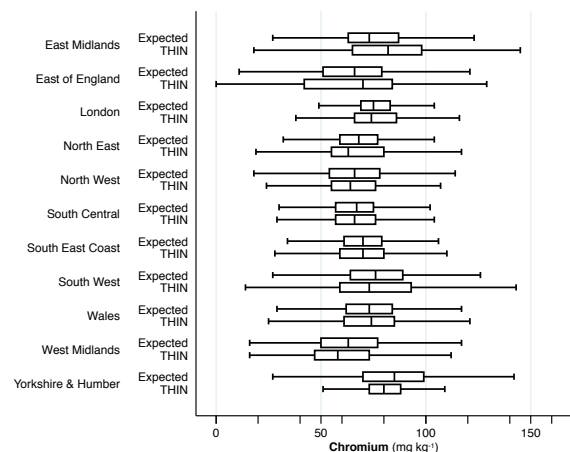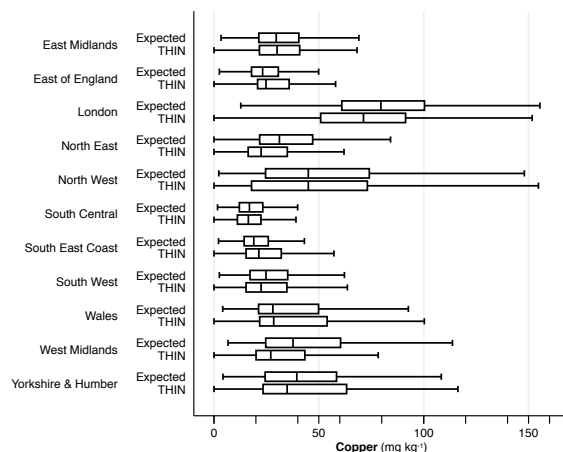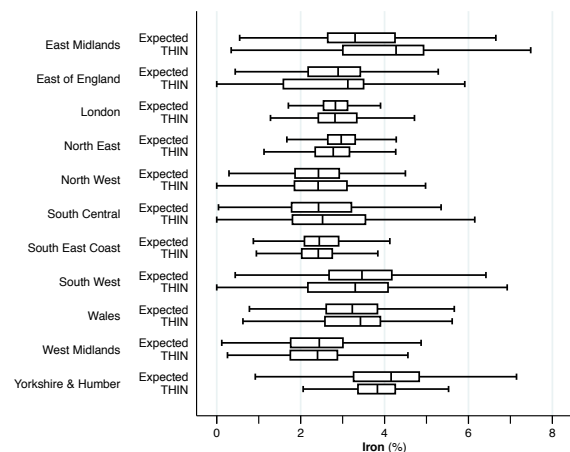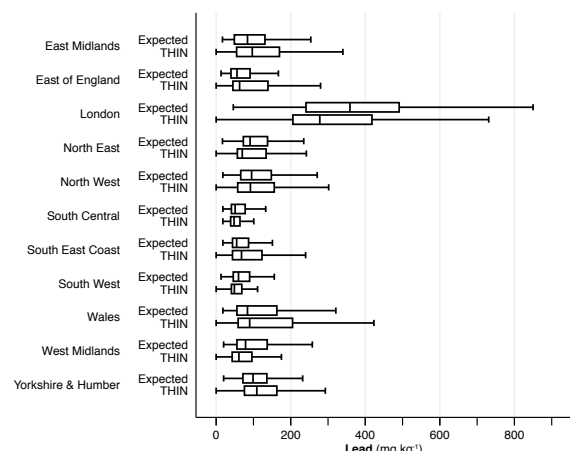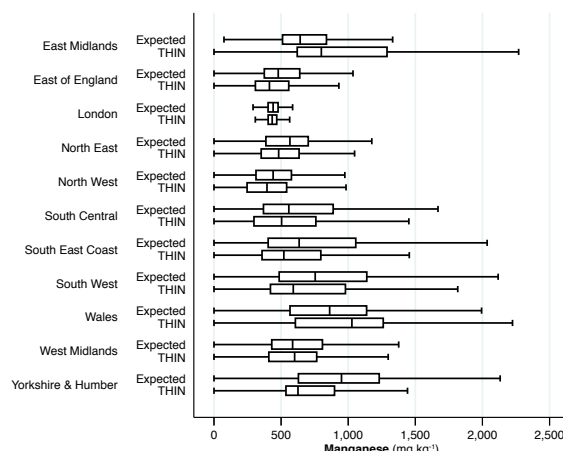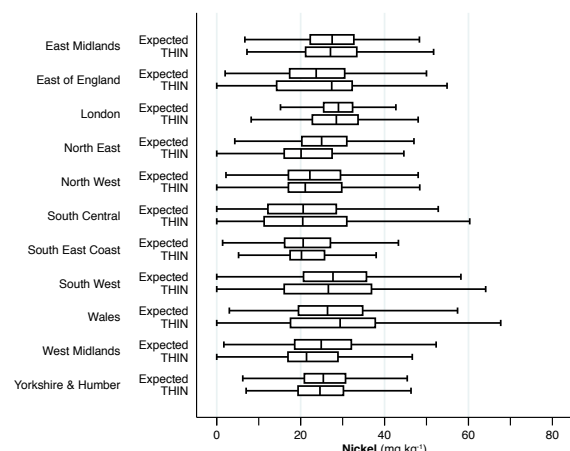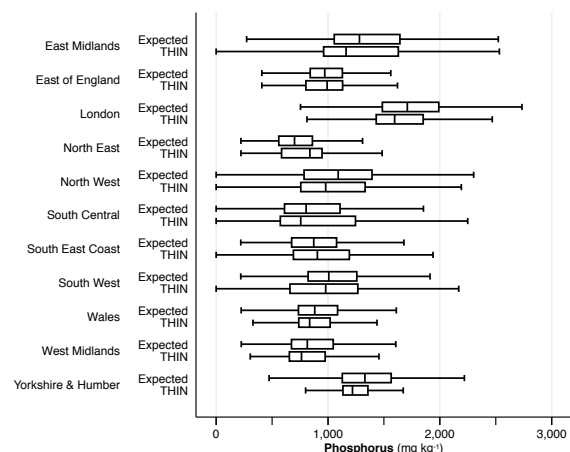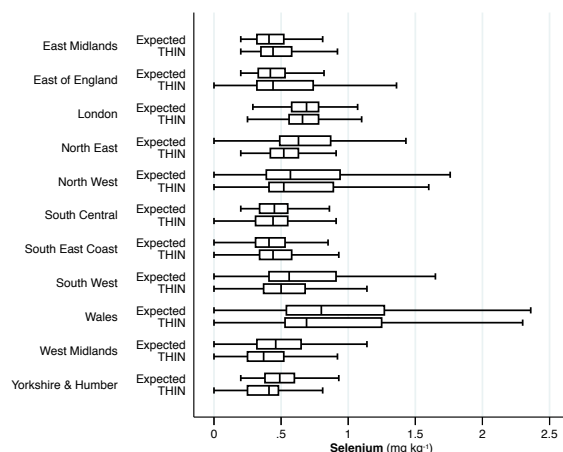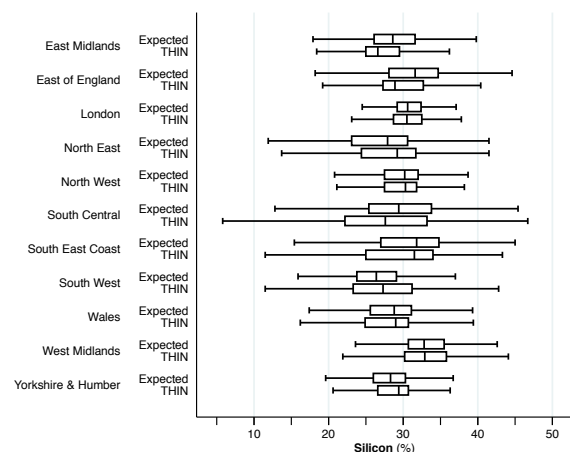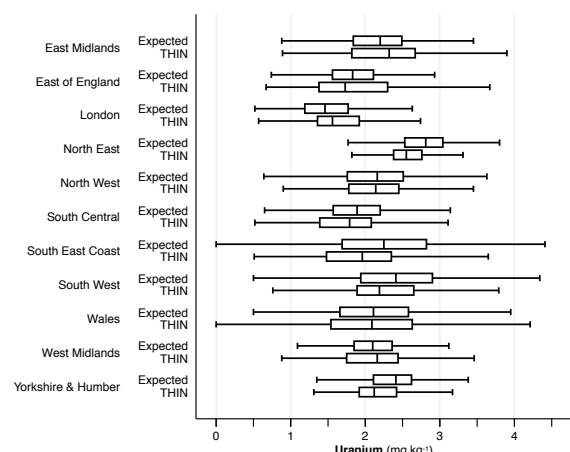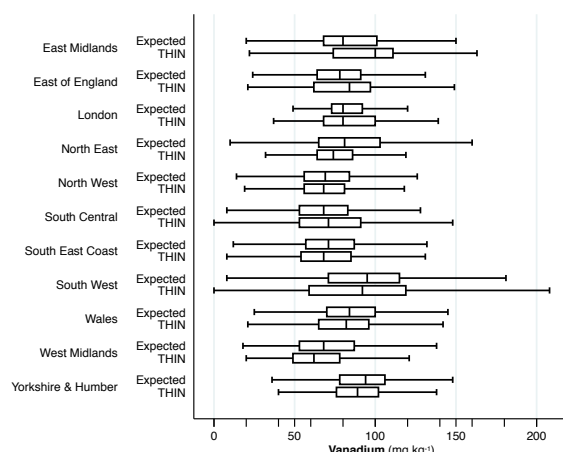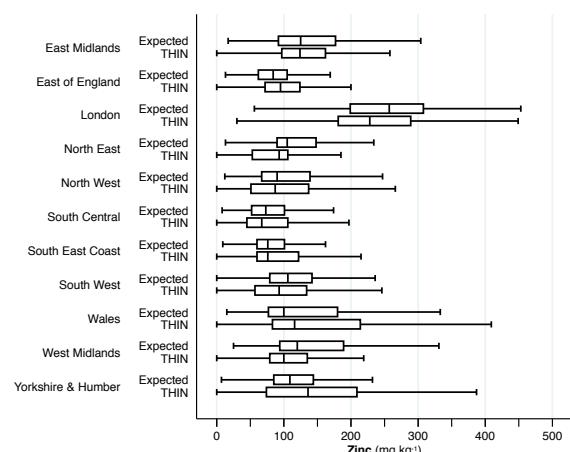

Supplement: Supplementary file 2 — Observed range of concentrations of 15 linked constituent elements in the residential soils of patients enrolled in The Health Improvement Network database on the date of the 2011 UK census, by Strategic Health Authority, and comparison with the expected range of concentrations in the residential soils of the entire Strategic Health Authority populations, estimated using 2011 Census population distributions. Boxes indicate interquartile ranges, midlines indicate median values and whiskers are drawn to the upper and lower adjacent values. (PDF 87 kb) [file 12963_2018_168_MOESM2_ESM.pdf]
